# Prediagnostic Plasma Metabolomic Profiles Using NMR for Exfoliation Glaucoma Among US Health Professionals

**DOI:** 10.3390/metabo15070469

**Published:** 2025-07-09

**Authors:** Akiko Hanyuda, Oana A. Zeleznik, Yoshihiko Raita, Danielle E. Haslam, Qi Sun, Kazuno Negishi, Louis R. Pasquale, Jessica Lasky-Su, Janey L. Wiggs, Jae H. Kang

**Affiliations:** 1 Department of Ophthalmology, Keio University School of Medicine, Tokyo 160-8582, Japan; akihanyu@keio.jp (A.H.); kazunonegishi@keio.jp (K.N.); 2 Epidemiology and Prevention Group, Center for Public Health Sciences, National Cancer Center, Tokyo 104-0045, Japan; 3 Channing Division of Network Medicine, Department of Medicine, Brigham and Women’s Hospital and Harvard Medical School, Boston, MA 02115, USA; nhotz@channing.harvard.edu (O.A.Z.); nhdah@channing.harvard.edu (D.E.H.); qisun@hsph.harvard.edu (Q.S.); rejas@channing.harvard.edu (J.L.-S.); 4 Department of Nephrology, Okinawa Prefectural Chubu Hospital, Naha 904-2293, Japan; yraita1@alumni.jh.edu; 5 GlaxoSmithKline K.K., 1-8-1 Akasaka, Minato-ku, Tokyo 107-0052, Japan; 6 Department of Nutrition, Harvard T.H. Chan School of Public Health, Boston, MA 02115, USA; 7 Department of Ophthalmology, Icahn School of Medicine at Mount Sinai, New York, NY 10029, USA; louis.pasquale@mssm.edu; 8 Department of Ophthalmology, Harvard Medical School, Massachusetts Eye and Ear Infirmary, Boston, MA 02114, USA; janey_wiggs@meei.harvard.edu

**Keywords:** exfoliation glaucoma, metabolomics, nuclear magnetic resonance spectrometry, omics

## Abstract

Background: Exfoliation glaucoma (XFG) represents a form of deleterious ocular aging of unclear etiology. We evaluated prediagnostic nuclear magnetic resonance (NMR)-based metabolites in relation to XFG risk, expanding on our prior findings of XFG-related metabotypes using liquid chromatography-mass spectrometry (LC-MS). Methods: We identified 217 XFG cases and 217 matched controls nested within three prospective health professional cohorts with plasma collected a mean 11.8 years before case identification. Plasma metabolites were analyzed using the targeted NMR Nightingale platform. Conditional logistic models and Metabolite Set Enrichment Analysis were performed. Multiple comparison issues were addressed using the number of effective tests (NEF) and false discovery rate (FDR). Results: Among 235 profiled metabolites, higher glucose was significantly associated with a lower risk of XFG (odds ratio (95%CI) = 0.42 (0.26, 0.7); NEF = 0.03). Among metabolite classes, lipoprotein subclasses and branched-chain amino acids were inversely associated, while relative lipoprotein lipid concentrations were adversely associated (FDR < 0.05). Conclusion: NMR profiling revealed that glucose, branched-chain amino acids, lipoprotein subclasses, and relative lipoprotein lipid concentrations may play important roles in XFG etiology.

## 1. Introduction

Exfoliation syndrome (XFS) is a systemic, strongly age-related fibrillopathy affecting an estimated 60–70 million individuals globally [1,2]. Approximately 10% of individuals with XFS progress to exfoliation glaucoma (XFG), the leading identifiable cause of secondary open-angle glaucoma [1]. The pathophysiology of XFG involves stress-induced elastosis, characterized by overproduction and abnormal cross-linking of microfibrillar aggregates, collectively known as exfoliation material [3]. These aggregates are deposited within the intertrabecular spaces, juxtacanalicular meshwork, and beneath the endothelial lining of Schlemm’s canal, resulting in increased intraocular pressure (IOP) and subsequent glaucomatous optic neuropathy [3]. The development of XFG is attributed to a multifaceted etiology involving age-related structural changes and a complex interplay between genetic susceptibility and environmental influences [4,5,6,7,8,9,10]. While a meta-analysis suggests an adverse relationship between cardiovascular disease and XFS [11], the studies comprising the data synthesis have limitations, and evidence indicates that XFS has a null association with cardiovascular mortality [12].

Advances in high-throughput “omics” technologies have revolutionized the understanding of the pathophysiology of multifactorial diseases, including XFG [13,14,15,16,17,18,19,20,21]. Genome-wide association studies (GWAS) have identified key genetic variants in *LOXL1* (lysyl oxidase-like 1) [13] and *CACNA1A* (α1 subunit of the type P/Q voltage-dependent calcium channel) [16], which are strongly associated with XFS. Complementary transcriptomic and proteomic investigations have revealed dysregulated expression of elastic microfibril components, including *LOXL1*, fibulin-2, and fibronectin, in the aqueous humor of XFG [14,15].

Further downstream in the “omics” cascade, metabolomics provides a functional readout of cellular activity, reflecting end-products of gene-environment interactions. Despite its potential, metabolomic studies in XFG remain sparse and often constrained by limited sample sizes (*n* < 35 for XFS cases). In a large case-control study (*n* = 205 XFG cases) [19], we utilized liquid chromatography-tandem mass spectrometry (LC-MS; Broad Institute, USA) to identify significant alterations in lipid metabolites, such as lysophosphatidylcholines and triacylglycerols, detectable in plasma up to a decade before XFG diagnosis [19]. Moreover, a recent lipidomic study (*n* = 9 for XFS, *n* = 14 for XFG) suggested elevated levels of cholesterol esters, phosphatidylcholines, triglycerides, and ceramides in the aqueous humor of XFG patients [20]. These findings underscore the critical need for large-scale metabolomics studies, particularly those enriched in lipidomic profiling, to further elucidate the metabolic mechanisms driving XFG pathogenesis and to identify potential biomarkers for early diagnosis and therapeutic targets.

Epidemiologic studies have extensively studied lipid metabolites and glaucoma, yet the findings remain inconclusive, particularly for XFG. Observational studies, including a meta-analysis, have predominantly identified a positive relation between plasma lipid levels and IOP [22,23]. However, the susceptibility to primary open-angle glaucoma (POAG) appears to vary based on specific lipid profiles: adverse associations with total cholesterol or triglycerols [23,24,25], protective associations with small-particle high-density lipoprotein (HDL; approximately 8.7 nm in size) [26], and null associations with other HDL and low-density lipoprotein (LDL) subtypes [26,27]. For XFG, the evidence is notably sparse and inconsistent, with prior studies reporting positive [28,29,30] or inverse associations [19] between triglyceride levels and disease risk.

Given the inconsistent findings in the existing literature, we followed up on our previous study using LC-MS [19] with the present metabolomic study of XFG, where we analyzed a panel of 235 prediagnostic plasma metabolites (the majority of which were lipids) measured by nuclear magnetic resonance (NMR) spectrometry, which excels in absolute quantification of low molecular metabolites and lipoprotein subtypes [31] as well as provides highly reproducible and quantitative data, from 217 XFG cases and 217 controls.

## 2. Materials and Methods

### 2.1. Study Design and Population

A nested case-control study was conducted within the three large US population-based cohorts (Nurses’ Health Study [NHS], NHS2, and the Health Professionals Follow-up Study [HPFS]). The NHS began in 1976 with 121,700 female nurses aged 30 to 55 years, and the NHS2 was initiated in 1989 with 116,429 female nurses 24 to 44 years of age at enrollment [32]. The HPFS began in 1986 with 51,529 male health professionals aged 40 to 75 years [33]. We collected standardized biennial questionnaires on diet, lifestyle, and disease outcomes such as exfoliation glaucoma (XFG) on these three cohorts with a >85% follow-up rate.

In our primary analyses, we have combined the XFG and XFG suspect (XFGS) cases, collectively referred to as “XFG” to increase the statistical power. To confirm incident XFG cases, we first identified participants who self-reported a physician diagnosis of glaucoma and obtained consent to access their medical records from eye care providers. A glaucoma specialist (L.R.P.) systematically reviewed all available visual field (VF) reports and glaucoma-related questionnaires completed by diagnosing clinicians or extracted from medical records. This review included an assessment of whether exfoliation material was present, alternative secondary causes of elevated IOP, maximum untreated IOP, optic nerve characteristics, filtration apparatus status, history of ophthalmic surgeries, and the earliest date of VF loss. XFG cases were defined by the presence of exfoliation material observed on slit lamp examination in conjunction with at least one of the following criteria: (1) maximum untreated IOP ≥ 22 mmHg, (2) cup-to-disc ratio ≥ 0.6 or an inter-eye difference ≥ 0.2, or (3) one or more reliable VF tests demonstrating glaucomatous damage. Exclusion criteria were the presence of exfoliation material without any glaucomatous signs (e.g., VF loss, elevated IOP, or abnormal cup-to-disc ratio) to minimize detection bias and restrict analysis to clinically significant XFS; a cancer diagnosis preceding XFG identification; and incomplete metabolomics data.

Blood samples were collected from 32,826 NHS participants between 1989 and 1990 (using heparin tubes), 29,611 NHS2 participants between 1996 and 1999 (heparin tubes), and 18,159 HPFS participants between 1993 and 1995 (EDTA tubes). Participants shipped their samples to the laboratory via overnight courier [34]. Upon receipt, samples were processed, and plasma aliquots were stored in liquid nitrogen freezers (≤−130 °C) [35]. Incident XFG cases were identified after blood collection through 1 June 2016 (NHS and NHSII), or 1 January 2016 (HPFS). Each case was matched 1:1 to a control based on age, cohort/sex, month and year of blood collection, time of day of blood draw, fasting status (≤8 h vs. >8 h), and race/ethnicity (Scandinavian, Southern European, other European, others). For female participants, matching also considered menopausal status and hormone therapy use at the time of blood collection (premenopausal, postmenopausal on hormone therapy, postmenopausal without hormone therapy, or missing/unknown) as well as menopausal status and hormone therapy use at the time of glaucoma diagnosis. Matching factors and covariates were obtained from questionnaires completed at the time of blood collection; when unavailable, data from the most recent prior biennial questionnaires were used.

The study protocol was approved by the institutional review boards of Mass Eye and Ear, Brigham and Women’s Hospital, Harvard T.H. Chan School of Public Health, and Icahn School of Medicine at Mount Sinai. This research was conducted in accordance with the principles outlined in the Declaration of Helsinki.

### 2.2. Metabolite Profiling

We submitted heparin (NHS, NHS2) and EDTA (HPFS) plasma samples for targeted high-throughput NMR metabolomics on the Nightingale platform (Nightingale Health Ltd.; Helsinki, Finland) [36]. In contrast to LC-MS, 1H NMR spectroscopy produces distinct spectral patterns for molecules containing hydrogen (H) atoms, where the area under each curve is directly proportional to the concentration of the corresponding molecule [37]. These spectral features are determined by chemical shifts and J-coupling patterns, which are derived from quantum mechanical principles, thus enabling precise quantification [31]. The platform facilitated the quantification of 249 metabolic measures, including routine clinical lipids (with 37 biomarkers certified for diagnostic use in the European Union), lipoprotein subclasses, fatty acid composition, and various low-molecular-weight metabolites, including glycolysis intermediates, ketone bodies, and amino acids, all expressed in molar concentration units. Of the 249 measures, 168 corresponded to the concentrations of specific metabolites, while the remaining 81 measures included ratios between metabolites, percentages of individual metabolites within total classes, or measures of unsaturation. Metabolites were categorized into 15 classes containing at least three metabolites being selected for evaluation: amino acids, triglycerides, lipoprotein subclasses, phospholipids, lipoprotein particle concentrations, total lipids, cholesterol, other lipids, free cholesterol, fatty acids, cholesteryl esters, lipoprotein particle sizes, glycolysis-related metabolites, relative lipoprotein lipid concentrations, apolipoproteins, and ketone bodies.

### 2.3. Pilot Studies Examining the Stability and Reproducibility of the Metabolite Measures

For 250 metabolic measures (249 metabolites plus glycerol, which was not assayed for the XFG case-control study), we conducted three pilot studies to evaluate (1) inter-assay reproducibility; (2) reproducibility with delayed processing of blood samples; and (3) within-person reproducibility over time. For inter-assay reproducibility, metabolites were measured in duplicate samples from eight heparin-collected participant plasma samples, eight EDTA-collected participant plasma samples, and two QC pools. Overall, the inter-assay reproducibility was good for most of the metabolites measured (Table S1). In heparin samples, the mean coefficient of variation (CV) was 7.2%, and 92% of the metabolites had CVs < 20%. In EDTA samples, the mean CV was 3.6%, and 98% of the metabolites had CVs < 20%.

For the delayed processing pilot, we included metabolites that were measured in triplicate samples processed and then frozen at ≤−130 °C at three time points (immediately, 24 hours (h), and 48 h later) among 7 heparin-collected samples and 7 EDTA-collected samples. Across all metabolites, the mean (max, min) intraclass correlation coefficient (ICC), Spearman’s rho 0–24 h, and Spearman’s rho 0–48 h were 0.80 (0, 0.99), 0.86 (0.13, 1.00), and 0.81 (−0.26, 1.00), respectively. In total, 94.4% of the metabolites displayed good reproducibility with an ICC or Spearman’s rho of ≥0.75 (Table S2). Of note, one metabolite class (the ketone bodies) had a 50% pass rate, where two of the four assays did not meet the above minimum threshold (amino acids and glycolysis-related metabolites), which were included in the primary analysis; overall, 14 of 249 metabolites failed the delayed processing pilot, and we excluded them for the XFG case-control study, leaving 235 metabolites for analyses.

For within-person reproducibility over time, the ICC and Spearman’s rho were calculated for metabolite concentrations measured in plasma samples collected 1–2 years apart among 40 NHS participants. The mean ICC and r were 0.70 and 0.64, respectively. In total, 92% of the metabolites displayed an ICC or r ≥ 0.40, indicating good reproducibility over time for most metabolites (Table S3).

### 2.4. Statistical Analysis

For clinical markers, we first truncated outliers to physiological concentrations (i.e., imputed them to the nearest physiological concentrations). For others, we imputed metabolites (*n* = 34) with missing values in <25% of participants using half the minimum value for each metabolite following methods described in our previous LC-MS study [25]. Also, because of the skewed distribution of metabolites, we transformed values to probit scores. This allowed us to evaluate associations with each 1 standard deviation increase for all metabolites.

We first conducted a case-control analysis where we used nested multivariable-adjusted conditional logistic regression analyses to identify specific metabolites associated with XFG. Several models with different covariate adjustments were conducted. In model 1, we included only matching factors such as age, sex, month/year/time of blood draw, and fasting status. In model 2, we further adjusted for smoking status (never, past, current), body mass index (BMI, kg/m^2^), and physical activity (metabolic equivalents of task-hours/week). In model 3, we added established XFG risk factors such as glaucoma family history [13], ancestry (Scandinavian ancestry, other European, others), time spent outdoors in sunlight in the summer in youth [38], history of non-melanoma skin cancer [39], latitude of residence [2], and residential population density [40]. In model 4, we further included modifiable XFG risk factors: intakes of folate [7] or caffeine [41], alcohol consumption [42], and total caloric intake. In model 5, we further added comorbidities related to XFG: stroke, heart disease, hypertension, high cholesterol, diabetes, hearing loss, and sleep duration [43]. In model 6, we further added use of drugs associated with glaucoma risk, including oral or inhaled steroids, as of blood draw [44].

Odds ratios (ORs) and 95% confidence intervals (CIs) were estimated for each unit increase in individual metabolite levels. To address the issue of multiple comparisons, adjustments were performed using the number of effective tests (NEF) [45], accounting for the high correlation structure inherent in the metabolomics data. Metabolites with an NEF less than 0.05 were considered statistically significant, whereas those with an NEF below 0.2 were regarded as potential candidates for further investigation, given the exploratory nature of the analysis. For metabolite class analyses, we used Metabolite Set Enrichment Analysis (MSEA) [46] first among 15 broad metabolite classes and then again among finer groups of 29 metabolite subclasses. In MSEA, the effect size of a metabolite class is quantified using a normalized enrichment score (NES). The NES reflects the extent to which a group of metabolites is disproportionately represented at the extremes of the ranked list of all measured metabolites—either at the top (metabolites with positive effect estimates) or the bottom (metabolites with negative effect estimates)—based on their observed effect estimates. The NES is adjusted to account for the size of the metabolite set. Since metabolite classes are generally uncorrelated, the false discovery rate (FDR) [47] was applied to correct for multiple comparisons, with an FDR < 0.05 deemed statistically significant and FDR < 0.2 denoting potential associations warranting further study.

In secondary analyses, we evaluated associations stratified by sex. In analyses by subtypes defined by XFG severity, we conducted analyses separately for two XFG subtypes: XFG (with VF loss; *n* = 100) and XFGS (without VF loss but with either elevated IOP or abnormal cup-to-disc ratio in either eye; *n* = 117).

All analyses were performed with SAS 9.4 (SAS Institute, Cary, NC, USA) and R 3.4.1 (R Foundation for Statistical Computing, Vienna, Austria).

## 3. Results

### 3.1. Characteristics of XFG Cases and Matched Controls

We identified 217 incident XFG cases (186 women and 31 men) and 217 matched controls. Compared with controls, XFG cases were more likely to have a family history of glaucoma and non-melanoma skin cancer, have higher intakes of caffeine, and experience outdoor sunlight exposure during summers in youth (Table 1).

### 3.2. Individual Metabolite Profiles

**Figure 1:**
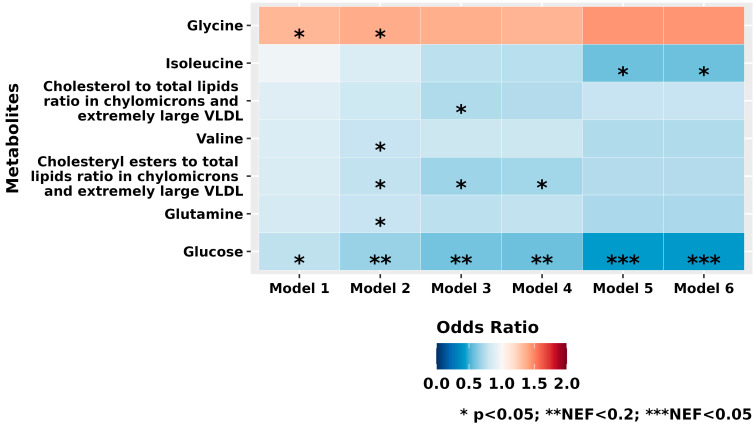
Individual metabolites among the *n* = 235 metabolites evaluated that were nominally significant (*p* < 0.05) across at least one of the various nested multiple conditional logistic regression models of exfoliation glaucoma (217 cases and 217 controls). Model 1 (basic model): Adjusted for matching factors only. Model 2 (metabolite-influencing factors): Model 1 plus adjustment for age, sex, smoking status, body mass index (BMI), physical activity, time and month of blood draw, and fasting status at the time of blood collection. Model 3 (established glaucoma risk factors): Model 2 additionally adjusted for type of European ancestry, family history of glaucoma, time spent outdoors in summer sunlight during youth, history of non-melanoma skin cancer, geographic latitude, and population density. Model 4 (homocysteine-related dietary factors): Model 3 plus dietary intake of folate, caffeine, alcohol, and total calories. Model 5 (comorbid conditions): Model 4 plus history of cardiovascular disease (heart disease, stroke), diabetes, hypertension, hypercholesterolemia, hearing loss, and sleep duration. Model 6 (glaucoma-related medication use): Model 5 plus systemic steroid use. * *p* < 0.05, ** NEF < 0.2, *** NEF < 0.05. Abbreviation: BMI, body mass index.

Among 235 plasma metabolites (Figure 1 and Table S4), we observed that glucose was significantly inversely associated with XFG. Although the significance was nominal in Model 1, in multivariable-adjusted Models 5 and 6, the results were significant even after adjusting for multiple-testing comparisons (NEF < 0.05). Among the other metabolites with nominal significance across the various models (*p* < 0.05), glycine was positively associated, whereas glutamine, branched-chain amino acids (BCAA; isoleucine and valine), and the ratios of either cholesteryl esters or cholesterol to total lipids in chylomicrons and extremely large VLDL were inversely associated with XFG.

### 3.3. Metabolite Class and Subclass Analyses

**Figure 2:**
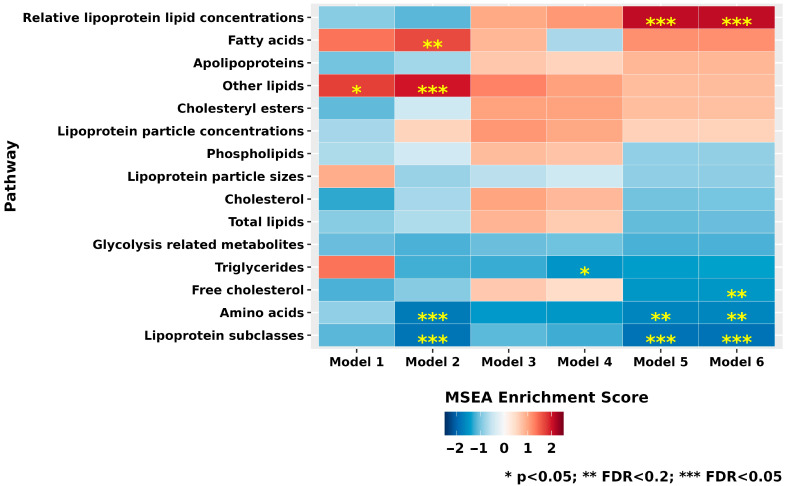
Metabolite groups (*n* = 15) evaluated in various nested multiple conditional logistic regression models of exfoliation glaucoma (217 cases and 217 controls). Model 1 (basic model): Adjusted for matching factors only. Model 2 (metabolite-related factors): Model 1 plus adjustment for age, sex, smoking status, body mass index (BMI), physical activity, time of day and month of blood draw, and fasting status. Model 3 (established risk factors for XFG): Model 2 plus European ancestry subgroup, family history of glaucoma, time spent outdoors in sunlight during youth, history of non-melanoma skin cancer, residential latitude, and population density. Model 4 (homocysteine-related factors): Model 3 plus dietary intake of folate, caffeine, alcohol, and total caloric intake. Model 5 (comorbid conditions): Model 4 plus presence of heart disease, stroke, diabetes, hypertension, hypercholesterolemia, hearing loss, and sleep duration. Model 6 (glaucoma-associated medications): Model 5 plus steroid use. * *p* < 0.05; ** False discovery rate (FDR) < 0.2; *** FDR < 0.05. Abbreviation: BMI, body mass index; MSEA, Metabolite Set Enrichment Analysis.

In MSEA of 15 broad metabolite classes (Figure 2 and Table S5), after multiple-testing corrections, in Model 6, we observed that the “lipoprotein subclasses” (top metabolite: cholesteryl esters in chylomicrons and extremely large VLDL) were significantly inversely associated with XFG, while the class of “relative lipoprotein lipid concentrations” (top metabolite: cholesterol to total lipids ratio in medium LDL) was significantly adversely associated with XFG (FDR < 0.05). The classes of “amino acids” (top metabolite: isoleucine) and “free cholesterol” (top metabolite: free cholesterol in VLDL) were inversely associated with XFG, at FDR < 0.2 level. Notably, the class of “other lipids” (top metabolite: ratio of triglycerides to phosphoglycerides) and “fatty acids” (top metabolite: ratio of omega-6 fatty acids to omega-3 fatty acids) were adversely associated in Model 2; however, these relations were no longer FDR significant in Model 6.

**Figure 3:**
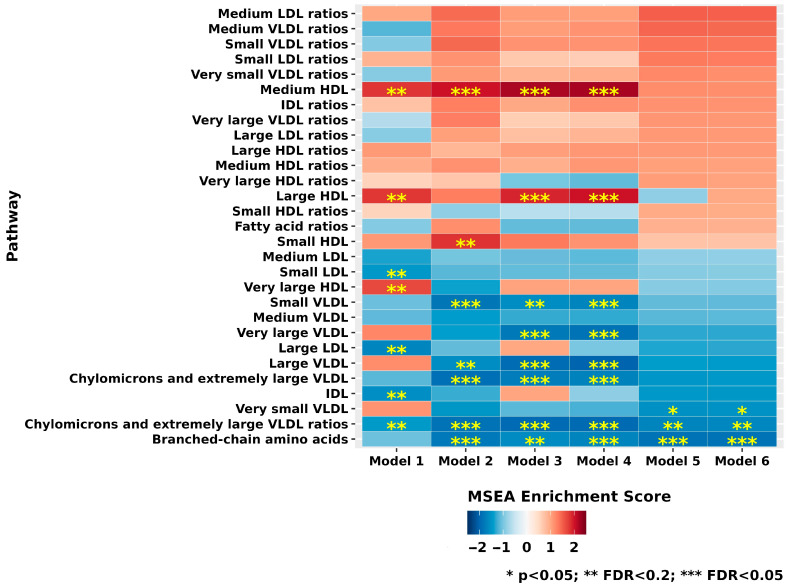
Metabolite subgroups (*n* = 29) evaluated in various nested multiple conditional logistic regression models of exfoliation glaucoma (217 cases and 217 controls). Model 1 (basic model): Adjusted for matching factors only. Model 2 (metabolite-related factors): Model 1 plus adjustment for variables known to influence metabolite levels, including age, sex, smoking status, body mass index (BMI), physical activity, time of day and month of blood draw, and fasting status at the time of blood collection. Model 3 (established risk factors for XFG): Model 2 further adjusted for type of European ancestry, family history of glaucoma, time spent outdoors in summer sunlight during youth, history of non-melanoma skin cancer, residential latitude, and population density. Model 4 (homocysteine-related dietary factors): Model 3 plus dietary intake of folate, caffeine, alcohol, and total caloric intake. Model 5 (relevant comorbidities): Model 4 plus history of heart disease, stroke, diabetes, hypertension, hypercholesterolemia, hearing loss, and average sleep duration. Model 6 (glaucoma-associated medication use): Model 5 plus systemic steroid use. * *p* < 0.05, ** FDR < 0.2, *** FDR < 0.05. Abbreviation: BMI, body mass index.

In MSEA analyses evaluating finer categories of 29 metabolite subgroups (Figure 3 and Table S6), we observed that in Model 6, branched-chain amino acids (top metabolite: isoleucine) was significantly inversely associated with XFG (FDR < 0.05). Chylomicrons and extremely large VLDL ratios (top metabolite: cholesteryl esters to total lipids ratio in chylomicrons and extremely large VLDL) were inversely associated with Model 6 at FDR < 0.2.

### 3.4. Stratified Analyses by Sex and Analyses by XFG Severity

In analyses stratified by sex (Figure S1), we observed that inverse associations with glucose was stronger in women, likely due to the much higher number of women than men. In separate analyses (Figure S2) for XFG (with VF loss) and XFGS (without VF loss), we observed that associations were in similar directions, with glucose showing stronger associations for XFG with VF loss.

## 4. Discussion

In this nested case-control study investigating prediagnostic plasma metabolites using a targeted NMR platform, we observed significant inverse associations with the metabolite of glucose, the metabolite class of “lipoprotein subclasses” and the metabolite subclass of branched-chain amino acids. We also observed significant adverse associations with relative lipoprotein lipid concentrations.

Our major finding was that higher glucose levels were associated with a lower XFG risk. To date, the relationship between hyperglycemia and XFG remains controversial [48,49]. Although a recent meta-analysis and systematic review of 14 observational studies (9 cross-sectional and 5 case-control studies) suggested no association between diabetes and XFS overall, there was a suggestive effect modification by age with a significant inverse association in those aged ≥ 65 years (OR = 0.71 [95% CI, 0.54–0.93]) [50]. Indeed, the pathophysiology of XFS/XFG is known to differ by age; while ocular trauma or surgical-related complications are major causes of XFS/XFG in younger populations [51], longstanding genetic and environmental interactions may result in XFS/XFG in elderly populations [4,5,6,7,8,9,10]. Chronic hyperglycemic status may cause increased advanced glycation end products in ocular tissues [52,53]. Of note, greater abnormal glycation of macromolecules or basement membrane components may prevent the formation of exfoliation materials in ocular tissues such as the trabecular meshwork, ciliary zonules, or lens capsule [4,54]. In our current study, the mean age was around 60 at the time of blood draw and 70 at diagnosis of XFG, supporting the inverse relationship between hyperglycemia and XFG.

We observed adverse associations with the metabolite class of relative lipoprotein lipid concentrations group. While the mechanisms underlying this association with XFG are not clear, this class of metabolites has been previously associated with benign eye and adnexa neoplasms [55], which include conditions closely linked to greater ocular UV exposure, further underscoring the potential role of UV exposure in XFG etiology [4,5,6,7,8,9,10].

Our results also confirm the associations of specific lipid and amino acid metabolites associated with higher IOP, which is a strong risk factor for XFG. For instance, our MSEA results suggested that lipoprotein subclasses, including chylomicrons and extremely large VLDL, were inversely associated with XFG. Interestingly, a metabolomics study using the NMR platform (*n* = 28,195 participants with 168 metabolites) in the UK Biobank reported differential associations between various lipids and IOP [56]; chylomicrons and extremely large VLDL as a class was associated with lower IOP [56].

Also, we observed inverse associations with BCAAs of isoleucine and valine, which is consistent with the significant inverse associations between BCAAs and IOP reported in a large-scale NMR metabolomics study (*n* = 28,195; −0.12 mmHg; NEF = 2.7 × 10^−5^) [56]. As XFG is characterized by elevated IOP, further studies are warranted to elucidate the metabolic pathways linking high IOP and XFG.

This study has several limitations. First, the study population consisted of US health professionals of non-Hispanic European descent, which limits the generalizability of our findings to populations with different racial and ethnic compositions. Second, blood samples were collected at a single time point and stored for several years, which may have resulted in sample degradation or artifacts due to interactions between metabolites and other molecules. In this case-control study, samples were matched based on the date of blood collection and were processed together for aliquoting and metabolomic analyses, minimizing potential biases, which, if present, would have attenuated the results towards the null. Furthermore, previous analyses using long-term stored NHS, NHS2, and HPFS samples have identified metabolites significantly associated with various health outcomes [57,58], with such results being replicated across multiple cohorts globally [59,60,61], suggesting that storage duration may have minimal effects on biomarker-disease associations. In the pilot study, some of the metabolites with high CVs (ketone bodies, amino acids, and glycolysis-related metabolites) may potentially produce greater noise. Residual confounding by unmeasured factors, such as social or environmental influences, remains possible despite our adjustment for several confounding variables, including dietary and seasonal variations, in multiple models. Moreover, there may have been minor nondifferential misclassification of the outcome, as controls (who did not have XFG) were not verified through medical records. Such misclassification should be minimal, as controls reported receiving an eye exam on the index match date, and the US prevalence of XFG is ~2.6% for those around 60 years [62], which would likely have biased the results toward the null. Due to the lack of external cohorts with prediagnostic blood collected, we lacked external replication of our current findings. Also, future studies should evaluate the metabolic trajectory before the onset of XFG in studies with longitudinal metabolomic profiling. Lastly, the current case-control study could be underpowered, even though it is the largest NMR metabolite study on XFG to date.

The strengths of this study are noteworthy. It includes a relatively large sample size (*n* = 217 cases and 217 controls) and profiled prediagnostic plasma metabolites approximately 10 years prior to the diagnosis of XFG using a rigorous NMR platform capable of analyzing an extensive panel of metabolites (*n* = 235). The study also benefits from rich covariate information, prospectively collected on lifestyle and clinical characteristics. Additionally, we conducted comprehensive analyses, including multiple sensitivity analyses in a case-control framework. These approaches provide novel insights into the metabolomic features associated with XFG pathophysiology.

## 5. Conclusions

In conclusion, in this study of prediagnostic plasma metabolites profiled by an NMR platform, we identified metabolites and metabolite classes associated with XFG: we observed significant inverse associations for glucose and BCAA and certain lipid ratios, along with the adverse associations noted for relative lipoprotein lipid ratios.

## Figures and Tables

**Table 1:** Characteristics of exfoliation glaucoma cases and their matched controls *.

Characteristics	XFG Cases (*n* = 217)	Controls (*n* = 217)	*p*-Value **
Female, *n* (%)	186 (85.7)	186 (85.7)	1.00
Mean age at blood draw (SD), years	58.6 (6.9)	58.0 (6.3)	0.40
Race/ethnicity, *n* (%)			0.30
Other European	166 (76.5)	152 (70.0)	
Non-European	2 (0.9)	1 (0.5)	
Southern European	34 (15.7)	49 (22.6)	
Scandinavian European	15 (6.9)	15 (6.9)	
Fasting >8 h, *n* (%)	149 (69.0)	182 (83.9)	<0.01
Mean age at diagnosis/index date of XFG (SD), years	70.4 (7.8)	69.8 (7.4)	0.47
Mean latitude of residence (SD), *°N*	39.7 (4.3)	39.6 (4.1)	0.92
Mean longitude of residence (SD), *°W*	−82.0 (13.3)	−82.2 (12.9)	0.91
Family history of glaucoma, *n* (%)	53 (24.9)	41 (19.4)	0.22
Mean caffeine intake (SD), mg/day	294.7 (244.4)	284.9 (246.7)	0.68
Mean folate intake (SD), mg/day	436.1 (227.3)	436.9 (249.0)	0.98
Outdoor sunlight exposure during summers in youth, *n* (%)			0.69
<1 h/week	17 (8.7)	15 (7.9)	
1–5 h/week	76 (38.8)	85 (44.5)	
6–10 h/week	64 (32.7)	59 (30.9)	
>11 h/week	39 (19.9)	32 (16.8)	
Non-melanoma skin cancer, *n* (%)	28 (12.9)	19 (8.8)	0.22
Mean alcohol intake (SD), g/day	6.8 (10.0)	6.3 (9.7)	0.59
Mean body mass index (SD), kg/m^2^	24.9 (4.4)	24.9 (4.2)	0.99
Smoking status, *n* (%)			0.29
Never smoker	104 (47.9)	110 (50.7)	
Past smoker	95 (43.8)	97 (44.7)	
Current smoker	18 (8.3)	10 (4.6)	
Mean physical activity (SD), MET-h/week	20.8 (24.7)	19.6 (21.4)	0.58
Hypertension, *n* (%)	3 (1.4)	8 (3.7)	0.22
Hyperlipidemia, *n* (%)	67 (30.9)	81 (37.3)	0.19
Diabetes, *n* (%)	5 (2.3)	6 (2.8)	1.00
Among female individuals:			0.96
Missing, *n* (%)	16 (8.6)	13 (7.0)	
Postmenopausal and current PMH use, *n* (%)	63 (33.9)	66 (35.5)	
Postmenopausal and no PMH use, *n* (%)	53 (28.5)	56 (30.1)	
Postmenopausal and past PMH use, *n* (%)	29 (15.6)	26 (14.0)	
Premenopausal, *n* (%) ***	25 (13.4)	25 (13.4)	

Continuous values are presented as the mean (SD) and categorical variables as *n* (%). * Blood samples were collected from female participants in the Nurses’ Health Study [NHS] during 1989–1990, from female participants in NHSII, and from male participants in the Health Professionals Follow-up Study (HPFS) during 1993–1995. ** Two-sided unadjusted *p*-values are reported. Group differences were assessed using the *t*-test, Wilcoxon rank-sum test, chi-squared test, or Fisher’s exact test, as appropriate. *** Analysis limited to women with complete information on menopausal status and use of menopausal hormone therapy. Abbreviations: h: hour; MET, metabolic equivalent of task; NHS, Nurses’ Health Study; PMH, postmenopausal hormones; SD, standard deviation; XFG, exfoliation glaucoma.

## Data Availability

Data from the health professional cohorts (NHS, NHSII, and HPFS) are not publicly available for the following reason: data contain information that could compromise research participant privacy. Reasonable requests to access these data can be made by vision research investigators one year after publication via http://www.nurseshealthstudy.org/researchers (accessed on 1 June 2025). Investigators can expect initial responses within 4 weeks of request submission.

## Supplementary Materials

The following supporting information can be downloaded at: https://www.mdpi.com/article/10.3390/metabo15070469/s1, Figure S1: Individual metabolites among the *n* = 235 metabolites evaluated that were nominally significant (*p* < 0.05) in Models 1 and 4 in strata of men (HPFS; *n* = 62) and women (NHS/NHS2; *n* = 372) in multiple conditional logistic regression models of exfoliation glaucoma (217 cases and 217 controls); Figure S2: Individual metabolites among the *n* = 235 metabolites evaluated that were nominally significant (*p* < 0.05) in Models 1 and 4 in analyses of subtypes of XFG defined by severity (XFG with VF loss or “EXF case” versus XFGS or “EXF suspect”) in multiple conditional logistic regression models of exfoliation glaucoma (217 cases and 217 controls). Table S1: Inter-assay (split pilot) coefficients of variation (CV) by anticoagulant type for metabolites measured with the Nightingale Metabolomics platform; Table S2: Delayed processing pilot: Spearman correlation coefficients and intraclass correlation coefficients (ICC) for metabolites measured with the Nightingale Metabolomics platform in EDTA and Heparin samples comparing samples processed immediately versus 24 h and immediately versus 48 h after collection; Table S3: Intraclass correlation coefficients (ICC) and Spearman (r) correlation coefficients for metabolites measured with the Nightingale Metabolomics platform in participant plasma samples collected 1–2 years apart (within-person stability); Table S4: Individual metabolites among the *n* = 235 metabolites evaluated that were nominally significant (*p* < 0.05) across the various nested multiple conditional logistic regression models of exfoliation glaucoma (217 cases and 217 controls); Table S5: Metabolite classes (*n* = 15) evaluated in various nested multiple conditional logistic regression models of exfoliation glaucoma (217 cases and 217 controls); Table S6: Metabolite subclasses (*n* = 29) evaluated in various nested multiple conditional logistic regression models of exfoliation glaucoma (217 cases and 217 controls).

## Abbreviations

The following abbreviations are used in this manuscript:

BCAA	Branched-chain amino acids
BMI	Body mass index
CI	Confidence interval
CV	Coefficient of variation
FDR	False discovery rate
GWAS	Genome-wide association studies
HDL	High-density lipoprotein
HPFS	Health Professionals Follow-up Study
ICC	Intraclass correlation coefficient
IDL	Intermediate-density lipoprotein
IOP	Intraocular pressure
LC-MS	Liquid chromatography tandem mass spectrometry
LDL	Low-density lipoprotein
LOXL1	Lysyl oxidase-like 1
MSEA	Metabolite Set Enrichment Analysis
NEF	Number of effective tests
NES	Normalized enrichment score
NHS	Nurses’ Health Study
NMR	Nuclear magnetic resonance
OR	Odds ratio
POAG	Primary open-angle glaucoma
SD	Standard deviation
VF	Visual field
VLDL	Very-low-density lipoprotein
XFG	Exfoliation glaucoma
XFS	Exfoliation syndrome
