# Supplementary material for: Prediagnostic Plasma Metabolomic Profiles Using NMR for Exfoliation Glaucoma Among US Health Professionals
Source: Metabolites. 2025 Jul 9;15(7):469. doi: 10.3390/metabo15070469 (PMC12299095; doi:10.3390/metabo15070469)
Supplement: Supplementary file 1 [file metabolites-15-00469-s001.zip › Supplementary Figures.pdf]

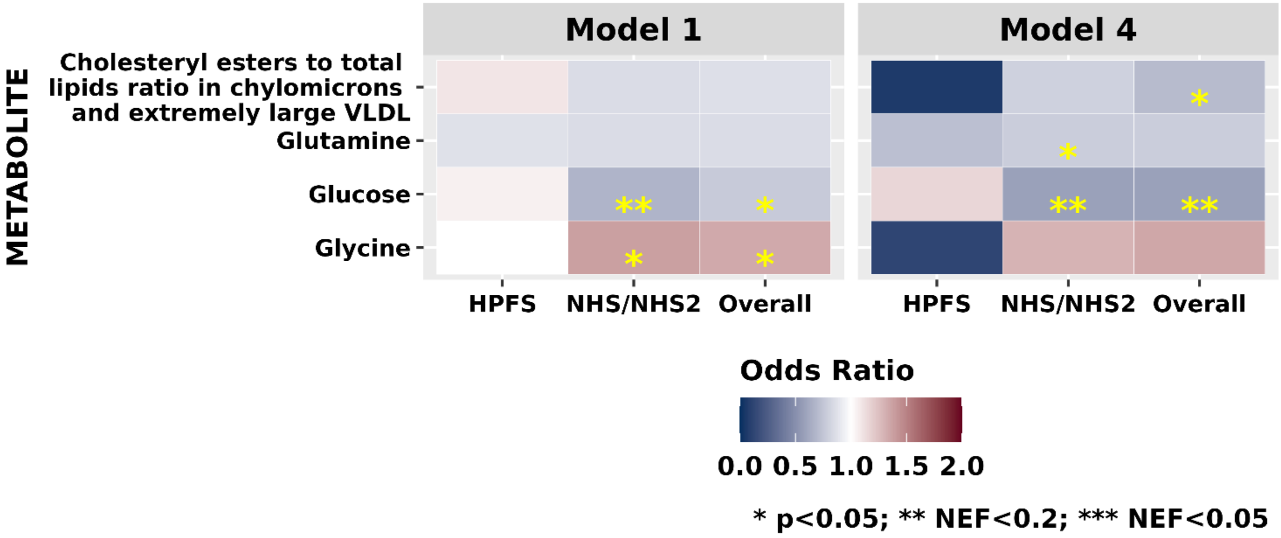

Figure S1. Individual metabolites among the n=235 metabolites evaluated that were nominally significant (p<0.05) in Models 1 and 4 in strata of men (HPFS; n=62) and women (NHS/NHS2; n=372) in multiple conditional logistic regression models of exfoliation glaucoma (217 cases and 217 controls)

Model 1: basic model, adjusting for matching factors only; **Model 4**: Model 1 adjusts for age, sex, smoking status, BMI, physical activity, time of day of blood draw, month of blood draw, fasting status at blood draw, type of Caucasian, family history of glaucoma, time spent outdoors in sunlight in the summer in youth, non-melanoma skin cancer, latitude, population density, folate intake, caffeine intake, alcohol intake, caloric intake.

* *P*<0.05, ** NEF<0.2, *** NEF<0.05.

Abbreviation: BMI, body mass index; HPFS, Health Professionals' Follow-up Study; NHS, Nurses' Health Study

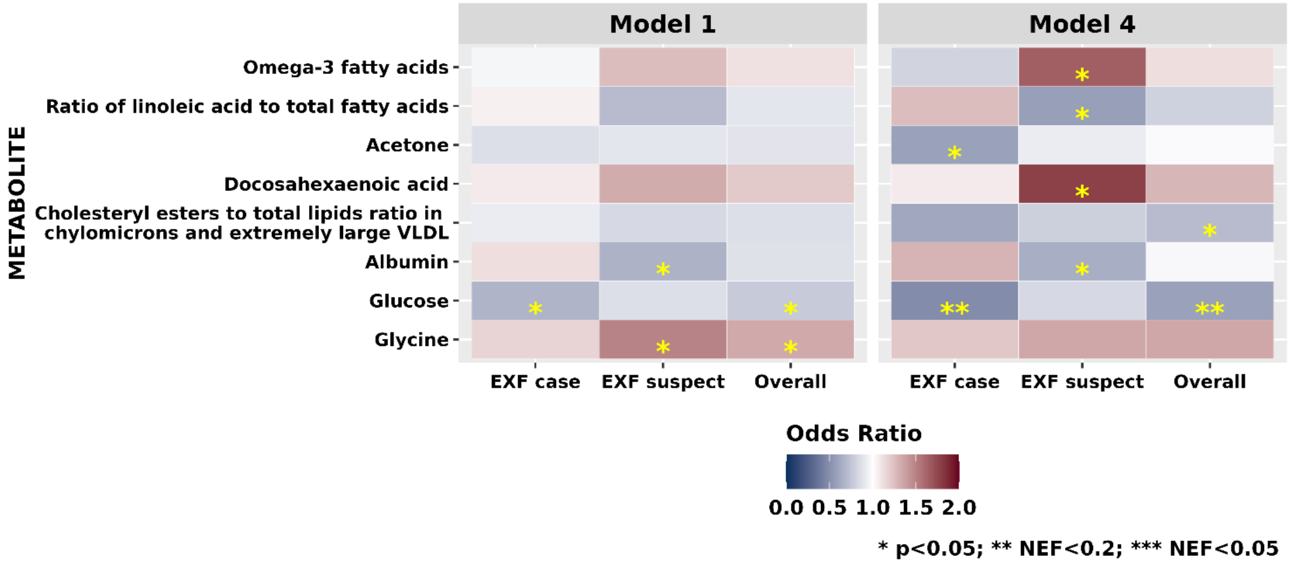

Figure S2. Individual metabolites among the n=235 metabolites evaluated that were nominally significant (p<0.05) in Models 1 and 4 in analyses of subtypes of XFG defined by severity (XFG with VF loss or "EXF case" versus XFGS or "EXF suspect") in multiple conditional logistic regression models of exfoliation glaucoma (217 cases and 217 controls)

Model 1: basic model, adjusting for matching factors only; **Model 4**: Model 1 adjusts for age, sex, smoking status, BMI, physical activity, time of day of blood draw, month of blood draw, fasting status at blood draw, type of Caucasian, family history of glaucoma, time spent outdoors in sunlight in the summer in youth, non-melanoma skin cancer, latitude, population density, folate intake, caffeine intake, alcohol intake, caloric intake

* *P*<0.05, ** NEF<0.2, *** NEF<0.05.

Abbreviation: BMI, body mass index; EXF, exfoliation; XFG, exfoliation glaucoma, XFGS, exfoliation glaucoma suspect
